# *Their* view: difficulties and challenges of patients and physicians in cross-cultural encounters and a medical ethics perspective

**DOI:** 10.1186/s12910-018-0311-4

**Published:** 2018-07-04

**Authors:** Kristina Würth, Wolf Langewitz, Stella Reiter-Theil, Sylvie Schuster

**Affiliations:** 1 Universitätsspital Basel, Hebelstrasse 2, CH-4031 Basel, Switzerland; 2 Psychosomatic Medicine - Medical communication, University Hospital Basle, Hebelstrasse 2, CH-4031 Basel, Switzerland; 3 Department Clinical Ethics, Psychiatric Hospitals University Basel University Hospital Basel / University of Basel, Wilhelm Klein-Str. 27, CH-4012 Basel, Switzerland

**Keywords:** Cross-cultural communication, Difficulties, Migrant patients, Ethics in clinical practice, Perspectives, Qualitative research

## Abstract

**Background:**

In todays’ super-diverse societies, communication and interaction in clinical encounters are increasingly shaped by linguistic, cultural, social and ethnic complexities. It is crucial to better understand the difficulties patients with migration background and healthcare professionals experience in their shared clinical encounters and to explore ethical aspects involved.

**Methods:**

We accompanied 32 migrant patients (16 of Albanian and Turkish origin each) during their medical encounters at two outpatient clinics using an ethnographic approach (participant observation and semi-structured interviews with patients and healthcare professionals). Overall, data of 34 interviews with patients and physicians on how they perceived their encounter and which difficulties they experienced are presented. We contrasted the perspectives on the difficult aspects and explore ethical questions surrounding the involved issues.

**Results:**

Patients and physicians describe similar problem areas, but they have diverging perspectives on them. Two main themes were identified by both patients and physicians: >patients’ behaviour in relation to doctors’ advice< and > relationship issues<.

**Conclusions:**

A deeper understanding of the difficulties and challenges that can arise in cross-cultural settings could be provided by bringing together healthcare professionals’ and patients’ perspectives on how a cross- cultural clinical encounter is perceived. Ethical aspects surrounding some of the difficulties could be highlighted and should get more attention in clinical practice and research.

## Background

Migrant health is on the agenda - in Switzerland and elsewhere [1]. The foreign population’s increase and its diversification [2] is reflected in Swiss healthcare settings, such as at the University Hospital Basel (USB) where ‘non-Swiss’ patients made up 35.6% of all outpatients [3] in 2011. Against the societal background of super-diversity [4, 5] communication and interaction in clinical encounters are increasingly shaped by linguistic, cultural, social and ethnic complexities.

Research on migrant health generally deals with differences between groups such as diverse migrant populations^1^ and respective local populations, for example in quality of care, access to care or on health outcomes [1, 6–9]. Yet, group attributions also hold difficulties and ambiguity as both, migrants, as well as respective local populations, are anything but homogenous social groups. However, literature shows that having a migration background is going along with risks to experience disadvantages regarding health and healthcare provision [6, 10], especially for migrants with a lower socioeconomic status [11].

Diverse aspects are mentioned in the literature as contributing to inequalities in health and healthcare between migrant- and local populations. Besides language barriers [12–14] the impact of ‘culture’ on cross-cultural communication and interaction has been shown [15–17]. Yet, vague and static cultural concepts, which “reduce individual behaviour to broad stereotypical formulas, or at least encourage such stereotyping,” [18] are still often to be observed in the medical context [18, 19].

At group level, ethical questions arise on issues such as the provision of equal opportunities in healthcare for all patients - a society’s duty to counterbalance inequalities which lead to health-disparities of diverse social groups [20]. Respective interventions promoted by governmental institutions (e.g. “National Strategy on Migration and Health” [21–23]), thus, aim at eliminating group-specific inequalities.

This study approaches the topic of migrant health in a different way: we were interested in the dimension of the individual and its ethical aspects: beyond the belonging to a specific societal group this dimension results from the immediate interaction with another unknown person. Research investigating clinical encounters between healthcare professionals and patients and comparing the perspectives on common communication and interaction has been done earlier [24] mostly focussing on different clinical areas or on diagnosis-related patient groups [25, 26].

Using an ethnographic approach, we investigated concrete experiences of patients of Turkish and Albanian origin and physicians they consulted with. The first author, a medical anthropologist, passively participated in their clinical encounters, observed communication and interaction (participant observation) and later interviewed patients and physicians about their perceptions of the encounter and the difficulties they had experienced. We provide views on the two most prominent categories of difficulties identified in the study: >patients’ behaviour in relation to doctors’ advice< and > relationship issues< and explore the surrounding ethical questions.

## Methods

This paper about difficulties and challenges is part of a larger ethnographic study (‘Hospital Ethnography at the University Hospital Basel’) on how patients and healthcare providers perceive their shared clinical encounters. We investigated issues influencing communication and interaction and tried to identify ethical aspects.

Based on hospital figures on outpatients and interpreter assignments, 16 patients of Turkish- and 16 of Albanian origin were recruited at the Medical Outpatient Clinic (MOC) and at Women’s Outpatient Clinic (WOC) of the USB. Ninety-four semi-structured interviews (patients and staff members) were conducted by the first author (KW) between August 2012 and January 2015. Several cycles of analysis on aspects relevant to the overall research question of the larger study (inductive formation of categories) resulted in the category set >difficulties and challenges<. The 34 semi-structured interviews in this category set have been used for this article (see Table 1). Interviews lasted up to 90 min, were tape-recorded and transcribed verbatim. The study was ethnographic in approach corresponding to the embedded research approach in medical ethics [27] and was approved by the Research Ethics Committee of North-Western and Central Switzerland (EKNZ).

**Table 1:** List of patients and doctors who were interviewed

Interview	Clinic	Role	Stated education	Years in CH	Subject of appointment
1	WC	Patient	Secondary school	11–20	Review Cysts
2	WC	Patient	Secondary school	0–10	Review Pregnancy
3	WC	Patient	N/A	21–30	Review Coil
4	WC	Patient	Secondary school	0–10	Annual routine review
5	WC	Patient	High school	21–30	Review Menopause
6	WC	Patient	Lower secondary school	11–20	Review Pregnancy
7	MC	Patient	N/A	21–30	Review Diabetes, hypertonia
8	MC	Patient	High school	21–30	Review Rheumatism
9	MC	Patient	N/A	11–20	Review Pain
10	MC	Patient	Lower secondary school	11–20	Review Rheumatism
11	MC	Patient	N/A	11–20	Review Hypertonia
12	MC	Patient	Secondary school	21–30	Review Hypertonia
13	MC	Patient	High school	N/A	Discussion lab results
14	MC	Patient	N/A	21–30	Review Hypertonia
15	WC	HCP	N/A	N/A	N/A
16	WC	HCP	N/A	N/A	N/A
17	WC	HCP	N/A	N/A	N/A
18	WC	HCP	N/A	N/A	N/A
19	WC	HCP	N/A	N/A	N/A
20	WC	HCP	N/A	N/A	N/A
21	WC	HCP	N/A	N/A	N/A
22	WC	HCP	N/A	N/A	N/A
23	WC	HCP	N/A	N/A	N/A
24	MC	HCP	N/A	N/A	N/A
25	MC	HCP	N/A	N/A	N/A
26^a^	MC	HCP	N/A	N/A	N/A
27	MC	HCP	N/A	N/A	N/A
28	MC	HCP	N/A	N/A	N/A
29	MC	HCP	N/A	N/A	N/A
30	MC	HCP	N/A	N/A	N/A
31^a^	MC	HCP	N/A	N/A	N/A
32	MC	HCP	N/A	N/A	N/A
33	MC	HCP	N/A	N/A	N/A
34	MC	HCP	N/A	N/A	N/A

^a^same HCP

Selection for recruitment was based on the list of patients scheduled for the next day if patients’ names suggested an Albanian or Turkish origin. At recruitment KW approached selected patients in the waiting area of the clinic, introduced herself as a PhD student conducting a study about cross-cultural communication. If patients had a follow- up appointment with the outpatient department (OPD), they were asked whether they would be willing to participate in the study. Patients with a Turkish/ Albanian sounding name but of non-Albanian/ non-Turkish origin (e.g. through marriage) and patients under 18 were excluded from the study. Written informed consent was obtained from all interview partners in German, Turkish or Albanian.

On the index day, KW met the patients in the entrance area of the respective clinic and accompanied them during all encounters they had with hospital staff (front desk staff, nurses, physicians and interpreters as required). Each involved staff member was also asked whether they were willing to be interviewed afterwards. (These data will be presented in another paper, here we report only on doctors’ and patients’ perspectives.) In general, patients’ interviews were conducted directly after consultation, staff members’ interviews were conducted as soon as possible after the interaction, usually the same week.

An observational grid served as a research instrument and for pre-structuring observations [15, 28–30]. It included aspects of verbal (e.g. conversation content, contribution to conversation), and non-verbal communication (e.g. mimicry, bearing) as well as interaction sequences according to social- (e.g. educational background) or culture-associated factors (e.g. regarding physical contact, gender). Relevant observations were noted in the interview guide. Besides participant observation during clinical encounters, KW performed general observations (e.g. front desk procedures) or held informal talks with staff and patients during long-time presence at both OPDs. After data collection, all observations were recorded in a field diary.

The semi-structured interviews combined a set of pre-defined questions developed from the literature study and questions that were triggered by the clinical experience of two practicing physicians in the research team (SCH & WL) in cross-cultural encounters. Interview questions were supplemented with observations KW had made during the clinical encounter under examination [15, 31]. Each interview covered sections on demographics, cultural and social aspects, language and communication. The interview guide was pilot tested during five interviews, discussed among the authors and revised accordingly. In the interview section that specifically addressed the index encounter, patients and doctors were first asked whether they themselves had experienced difficulties or challenges. Then, in line with the observational grid, observations made during the clinical encounter, were brought up. Language during interviews was based on the same conditions as during medical consultations: Most interviews were conducted in German, if patients had used an interpreter (professional or non-professional) during medical consultations, the same interpreter was also involved for the interviews.

Data of the semi-structured interviews were evaluated content-analytically according to Mayring [32–34] using the qualitative analysis software MAXQDA for coding and analysis. Observational data were primarily used during the interviews; all field notes (e.g. on observations during clinical encounters, drawings on the setting, general observations about the setting) were filed. Interviews were analysed simultaneously; from the beginning, commonalities and differences in patients’ and physicians’ perspectives were acknowledged. Interviews with coded text sequences in the category set >difficulties and challenges< were analysed further for respective content: significant interview sections were marked and discussed among the authors. Next, text sections were coded, reduced and verified in several cycles. Codes and sub-codes were developed inductively. Coded sections of patients’ and physicians’ interviews were compared, and codes were collated where appropriate (triangulation). Presented interview citations are labelled with the interview number according to Table 1.

## Results

The larger study including hospital staff, patients, nurses, and doctors, revealed diverse topics some of which were specific for different groups and others overlapping between them. For example, specifically in professional groups of administrative- and nursery staff, a major category entitled >professional stance< was formulated including personal attitudes and mind-sets towards migrant patients and migrants in general. For the purpose of this paper, we tried to identify topics that were common to patients and, at least, one of the other healthcare professional groups. There, a major issue common to patients and physicians concerned perceived difficulties. The respective category >difficulties< included two main problem areas: >patients’ behaviour in relation to doctors’ advice< and > relationship issues<.

### Patients’ behaviour in relation to doctors’ advice

Physicians reported of patients who took medication in wrong doses or at the wrong time, patients’ insufficient or lacking record of medical values (e.g. blood sugar values to adjust insulin treatment, or blood pressure values to control hypertonia) or of patients forgetting to bring these records to the consultation. Patients referred to these as well and mentioned difficulties in following the doctors’ suggestions, especially concerning medication, self-monitoring and adhering to follow- up appointments.

Both physicians and patients also offered some explanations why these behaviours occurred. These explanatory concepts will be described in detail in the following section.

#### Views of physicians

##### >deficiencies<

>Missing understanding of medical concepts<, >lack of basic medical knowledge< and > intellectual barriers<.

A physician talking about his frustration about a patient with gestational diabetes, who again did not bring the blood-sugar diary to the consultation, explained:

*“I think she hasn’t the basic [medical] overview […] very often, women do not see that high blood pressure has to do with the child and that blood sugar is connected to pregnancy. […]” (I 19).*

Another physician presuming similar reasons for her patient not following therapeutic instructions for losing weight additionally stated:

*“[…] maybe she is also intellectually not capable to understand how these medical facts are linked […].” (I 21)* and further guessed:

*“[…] not only laziness or habit hinders her, […] also she is not aware that she really could reduce her climacteric symptoms by losing weight.” (I 21).*

Dealing with non-fulfilment of medical recommendations was also linked to considerations about >responsibility<. For example, one physician concluded after a patient had not followed medical advice:

*“I am quite pragmatic here. The patient is 18 years old, he is responsible and obviously he isn’t neuro-cognitively disabled. […] This is no longer topic for me then.” (I 28).*

##### >ignoring medical reality<

>Fixation on own ideas<, >not seeing the seriousness< and < indifference towards one’s own health<.

A physician speaking about a patient with hypertension not measuring blood pressure at home and recording them in a diary assumed him to be:

*“[…] one of those patients with migration background […] having quite different ideas about how to look after health or what to do for it.” (I 27).*

Also, he admitted wondering to what extent this patient wished to be informed at all regarding improving his condition, saying:

*“Some […] informed patients, they also query by themselves […] and then you sense […] when you explain […] it falls on fruitful ground, they implement it. And with him I had the impression, even if I would engage in explaining once again, he cannot or doesn’t want to implement it.” (I 27).*

Other related explanations in this context are summarized under the categories ‘*disinterest’*, ‘*having other priorities than health’* or ‘*little suffering’*.

#### Views of patients

##### >being afraid of negative results<

Regarding self-monitoring issues, some patients with rather poor general living conditions (difficulties in the private field, additional health issues) revealed to be afraid of seeing their own negative results (e.g. high blood pressure values) because this would lead to even more distress (e.g. anxious thoughts). One patient explained his difficulties in taking the values at home:

*“You know, if you know about the risks [of high blood pressure] then you get upset. If you don’t know about the dangers of these diseases, then you can comfortably note [the values]. Yeah, you can measure five times and note it ten times. But I KNOW the dangers of hypertension, diabetes and other diseases.” (I 7).*

Another patient suffered from similar fears. When he was asked to measure blood pressure during consultation, he measured a second and third time on his own initiative. Afterwards he explained his reasoning behind this behaviour:

*“I hate this. […] When I see the blood pressure measuring device I always get afraid and then the value is automatically too high.” (I 12).*

and further revealed why he feared seeing high values:

*“I want to see that it [blood pressure level] is ok. Then I can go [home]. I don’t want to go home with one hundred fifty, one hundred sixty. [Because] then, I always wonder ‘hmmm? Why is this so?’, and again and again...” (P15).*

##### >social distress<

Distressing factors such as workplace-related issues or family issues were mentioned as common reason for not following medical advice. When asked for the reasons why he didn’t self-monitor blood pressure at home, one patient, working in two jobs, revealed:

*“When I [get up] in the morning, I am tired. When I come [home] in the evening, I am also tired, you know. [There is] Not much time.” (I 11).*

But later he added hoping for an improvement of his health situation after quitting the second job where he experienced particular distress because of his writing difficulties:

*“Now we have a fair […] I have a […] boss who is a little strict. He wants us all to take written notes. This provokes a little bit stress.” (I 11).*

Another patient revealed a difficult family situation and financial problems provoking distress and making it difficult to follow complex treatment instructions.

### Relationship issues

Both patients and physicians described experiencing difficulties at the level of their relationship. Physicians mentioned difficulties in perceiving the patient as a tangible person (e.g. “I cannot not FEEL what’s s/he like”), with demands of patients (e.g. when insisting on prescriptions), patients’ pain expression (e.g. difficulties in attributing expressed pain to physical symptoms) and with the role of male family members (e.g. husbands speaking for their wives). Patients felt not being taken seriously (e.g. when they perceived a physician’s response to their complaints as insufficient) or fobbed (e.g. when prescriptions in pain medication didn’t meet their expectations) and registered a lack of careful attention from physicians (e.g. when physicians didn’t know certain details from a patient’s record). Also, some patients reported a sense of discrimination that they attributed to their origin, their nationality, or their language [problems].

#### Views of physicians

##### Difficulties in perceiving the other as tangible person, reaching common ground

One physician tries to explain what in his view renders relationship-building with migrant patients more difficult compared to Swiss patients:

*“[…] I called her [a Swiss patient] because I had to know something. I just got the feeling I actually capture how she is, or rather is. And I can react on this. Or I also sense how I am perceived, if she understands me or if she takes my statements completely wrong, […] it is a bit about what’s in between the lines. This [feeling] I frequently experience as less present with migrant patients.” (I 29).*

Another physician explains his frustration with the distance he perceived during the encounter with a middle aged Albanian woman with joint pain:

*“[…] it was not the whole language issue that bothered me most, but rather how she is in her behaviour. I don’t know, she was very reserved; you really had difficulties in knowing what she thinks […]. There was a lot of distance.” (32).*

Later, he specified what he had been missing:

*“[…] how you are feeling, comfortable, uncomfortable why and so on. […] Everything that’s in your head but that you probably won’t say.” (I 32).*

##### Demanding patients, exaggeration of pain expression

Some physicians viewed patients’ behaviour as overly demanding with an imbalance between high claims towards physicians and low willingness to contribute themselves to improve their health. One physician specified:

*“[the patient] he comes with quite concrete claims and relatively low own activity. […] He needs a medical certificate, he needs - in his first consultation - he needs prescription for physiotherapy […] then he unexpectedly showed up in-between because he just got a little cold needing a recipe […] He always comes with a whole range of prescriptions [needed] but seldom [says] […] ‘*Would you have any suggestions of what *I* (!) can do*?’” (I 29).*

Some patients’ ways of expressing pain created challenges for many physicians particularly because they felt that patients’ > exaggerated the expression of their own suffering<; they found it extremely difficult to >get a grip on the pain< described, feeling uneasy to address it. For example, one physician assumed his patient had heavily overstated his rheumatic pain:

*“Based on our results I wasn’t able to reconstruct [his pain] from having difficulties walking up to [using] the wheelchair. And that is why I had the impression it was also a bit a primary gain for him.” (31).*

Another physician having similar difficulties in finding “objective” problems (e.g. “an inflammation”) in understanding his patient’s vociferous reactions on diverse moves during physical examination:

*“The problem is that it [the moves during physical examination] didn’t fit with her wailings. […] It wasn’t really connected [to the medical results].” (I 32).*

##### Role of family members

Physicians often perceived difficulties in communicating openly or directly with their patients. This held particularly true for some female patients’ in whom the presence of their husband was perceived as blocking an open exchange with the patient. One female physician describes her feelings of frustration:

*“There are some women, I think [..] if they were alone, they could speak more than just one word. And if the husbands are with them they don’t do so. […] For example, if I ask, ‘does your wife have this and that’ he is not asking her but just says ‘no’.” (I 19).*

#### Views of patients

##### >not being taken seriously<, >being fobbed< and > lacking attention<

The impression of not being taken seriously came up e.g. when a concern was not dealt with the way a patient had expected. One pregnant woman said:

*“I have always told [that I have headache]. But [she] always asks if I drink enough. […] As soon as I say I have headache the question comes up if I drink enough. I find this a little strange […] because I drink enough and she always says to me ‘[you have headache] because you must drink more.’” (I 2).*

Another patient alluded to a similar sense of neglect when she said:

*“they actually say I have nothing, they don’t find anything. But I also don’t know where the pain comes from. This is very difficult for me because I have pain, my belly is swollen and they don’t know where it comes from. […] A colleague of my mother came three times here, she complained about pain and they sent her home saying she had nothing at all. Finally, it came out that she had breast cancer.” (I 1).*

Some patients reported of experiences making them feel fobbed:

*“I had so much pain. […] If you go to the doctor because you need help and you say, ‘I have pain’ he gives you a pill without knowing what exactly you have. For example, I have problems with my joints and he gives me medication for flu.” (I 10).*

The sense of missing attention was also manifest in quotations like >s/he was not interested in me< or > s/he was not prepared for the encounter<. One patient who felt her physician had missed to catch up a specific point during consultation complained that she had not read the medical record properly even though she had had the time for doing so. She told the interviewer:

*“Although she has had the time [to read the medical record [written in German]], she waited [for the interpreter]. During this time, she should have read [the medical record] more thoroughly.” (I 3).*

### A sense of discrimination

Feelings of discrimination or being treated harshly were expressed and sometimes attributed to the patients’ origin, nationality or language. One patient spoke about her experiences with unfriendliness of staff members. When she was explicitly asked whether she thought that this was connected to her limited language skills, she admitted hesitantly:

*“Yes. […] Because you sense how one talks to you […]” (I 2).*

Another patient who felt rejected without having received medical help repeatedly was very clear on that matter:

*“[they might think] ‘anyway he won’t complain’, maybe because I am an Albanian. […] I really do think that way! You know, [they just think] ‘he won’t complain.’” (I 10).*

## Discussion

This study presents difficulties that migrant patients and physicians experienced in their cross-cultural clinical encounters and explores which ethical issues are connected with them.

The category >problems with patients’ behaviour in relation to doctors’ advice< included taking medication in wrong doses or at wrong times, insufficient or lacking record of blood sugar or blood pressure values or carrying respective records to the consultation. Physicians usually located reasons for these issues on the patients’ side, referring to shortcomings such as patients’ denial of >medical facts< or > lacking medical knowledge<. Patients reported struggling with such problems such as >being afraid of seeing their own negative results< or problems with regard to >social distress< (e.g. at their workplace). Comparing the patients’ and healthcare providers’ explanations on the reported issues reveals meaning differences. For example, a physician supposes his patient to be >fixated on own ideas< about the disease and therefore not following his medical advice. The same patient assumes that workplace distress is hindering him from following therapeutic instructions.

Using the process of change developed by Prochaska and Di Clemente [35–37] helps to acknowledge that doctors and patients seem to be aware of the problem, even to agree on the significance of finding common ground and reaching shared decisions about treatment and behaviour. Thus, the similarity of the description of problem areas is a promising finding in itself, showing that both parties are not indifferent, i.e. in the state of pre-contemplation [35]. However, when doctors and patients realise that they actually did not reach common ground, the strategies they apply do not prove successful. Some of the physicians’ explanations on the reasons why patients did not follow their suggestions are reflected in the scientific discourse on social determinants of health: contextual medical knowledge is based on educational background, a vulnerability factor deeply influencing health outcomes [11, 38]. This connection is also consistent with basic socio-demographic information provided on patients presented in this study (Table 1). Links between the physicians’ perceptions of patients, their origin and socio-economic status have been shown by van Ryn et al. as well as the observation that physicians tend to perceive patients with lower socio-economic status as less intelligent than others [39, 40].

Relationship issues reported by physicians included experiences of distance from- or inability to connect with their patients, demanding behaviour, patients’ pain expression and their experiences with the role of male family members. Relationship issues reported by patients included feelings of not being taken seriously or fobbed, lacking attention on behalf of physicians and feelings of discrimination. The comparison of reported problems raises questions on how these issues relate to each other and how they impact a clinical relationship (e.g. physicians’ experiences of distance occurring alongside patients’ frustrations of not being taken seriously). An important ethical aspect derives from the question of how shared decision- making can be ensured under such circumstances.

A large body of literature has shown that healthcare professionals and patients often encounter challenges in their interactions due to differences in language, culture or (religious) value systems [41–44]. From a medical ethics perspective, difficulties of migrant populations have particularly been discussed in the light of cultural difference and its implications for a principle- based approach [45], for example when the principle of autonomy collides with the wish to act culturally sensitive due to differences in (understanding of) moral values as shown by Minkhoff [46]. However, the problem of mutual misunderstandings is interesting insofar as it seems to indicate a fundamental incongruence at a level below cognitive differences or differences in values. Drawing from their experience with Swiss patients, doctors realise that resonance is often difficult to reach with patients from a migration background. Phenomenological theory offers the term >immediate impression< to describe what is missing here: an effortless and immediate sense of mutual understanding that cannot be derived from single observations like facial expression or prosody. “When we have such an immediate impression, we can almost instantly understand and enact the appropriate behavioural response.” [47]. As far as policy making is concerned, probably raising problem awareness is necessary first on both sides by introducing this concept. Immediate impression resembles the idea of “mentalising” [48] being defined as the ability to understand the mind of another person (and of oneself). Interestingly, some hospitals in Switzerland have realised that (even) doctors and nurses from Germany sometimes need a “sensitisation course” for working with Swiss colleagues and patients, as sharing a language is a necessary, but not sufficient condition to reach common ground.

Reducing the disadvantages of patients with migration background by implementing interventions to improve communication in cross-cultural encounters is intrinsically linked with the question of responsibility (for action). At higher level, institutions or policymakers adopt this task establishing health policies to reach that goal. Within the dimension of the individual encounter the question of responsibility is more complex. Usually, changes in behaviour are based on the perception of not just a difficulty but of a *problem.* For such a change in perspective – from simple difficulties to problems requiring action - one point of reference could be what Ahola-Launonen (2015) calls an “evolving idea of social responsibility in bioethics”. Some medical ethics approaches promote an individualistic view on health placing the individual’s responsibility for his/ her own health in the centre – quite similarly to the overall individualistic tradition of medical history. A physician’s comment on his patient not following medical advice mirrors this basic attitude perfectly “*The patient is 18 eighteen years old, he is responsible and obviously he isn’t neuro-cognitively disabled. […] This is no longer topic for me then.” (I 28)’*. Yet, against the proven background of social factors contributing to health [11, 41], a call for medical ethics to take the social context into account has been made, arguing that “holding individuals solely responsible for their own health is not a fair conclusion, because so many determinants of health are beyond the individual’s control” [49]. We assume that acknowledging this view may contribute to a climate in medicine where the social perspective can become an integral part of what is considered relevant for an individual’s health. On the individual level of encounter, such a shift in assumption helps to reduce the discrepancy which arises when regarding the patient as an “autonomous agent” [49] and on the other hand being confronted with his/ her health-related behaviour which is based on social or personal conditions.

But the issue of responsibility also arises in another respect: explanations of some physicians held on why patients did not follow medical advice seem to have something “ultimate” about it (e.g. assuming a patient does not follow treatment instructions because he/ she is fixated on own ideas is suggestive of being the end of efforts to solve this situation and find other ways to overcome barriers to effective treatment) and include questions about responsibility and duty of care towards a patient in need: (when) is it allowed to stop trying? The concept of professionalism which “requires that doctors adhere to certain principled responsibilities” [50] includes the responsibility to reach a shared understanding, in a sense that the patient makes an informed choice. Against the background of lacking resonance and difficulties in finding common ground, ethical shortcomings in the apparent absence of shared decision- making become obvious though.

### Limitations

Compared to quality criteria from quantitative research and on the basis of the interpretive paradigm, the study’s objective is not generalisation of results, but providing “a rich, contextualized understanding of some aspect of human experience through the intensive study of particular cases” [51]. The question whether our findings have any bearing beyond the specific setting of the USB, the hospital where the investigation took place, cannot be answered by a simple reply, but has to be put to discussion. Assuming that hospitals differ in their very communication culture, we cannot exclude that a similar investigation in another hospital would find different topics relevant. On the other hand, the most important areas of interest that are reported in this paper reflect those that have been mentioned in the literature. However, we used the strategy of triangulation by including several different data sources (patients and healthcare professionals) and using different data collection methods (interviews and observations) to ensure trustworthiness of our findings.

## Conclusion

Our innovative approach used to assess problems that arise with cross-cultural communication in medicine by combining participant observation of clinical encounters with reflection in doctors and patients’ accounts of what had occurred in these encounters, proved fruitful as it led to rich data stimulating reflection. This detailed approach points to the importance of concordance for both, patients and physicians, and the importance of clinical relationships. Taking the proven links between migration background and inequalities in health, insights provided are crucial to better understand what the difficulties on the individual level of encounters are and how medical ethics can contribute to a shift in perception, from simple difficulties to *problems* having real consequences. Similar studies should investigate clinical encounters with diverse patients in other settings or hospitals to further validate our results or identify additional issues associated to these.

## Abbreviations

EKNZ: Ethikkommission Nordwest- und Zentralschweiz
HCP: Healthcare professional
MC: Medical Outpatient Clinic
NCE: Swiss National Advisory Commission on Biomedical Ethics
USB: University Hospital Basel
WC: Women’s outpatient clinic

## Footnotes

1 The term population with a migration background as defined by the Federal Statistical Office (FSO) ‘includes all foreign nationals, naturalised Swiss citizens, except for those born in Switzerland and whose parents were both born in Switzerland, as well as Swiss citizens at birth whose parents were both born abroad’. (https://www.bfs.admin.ch/bfs/en/home/statistics/population/migration-integration/by-migration-status.html) Accessed 02 July 2018

## References

[1] van den Muijsenbergh M, van Weel-Baumgarten E, Burns N, O'Donnell C, Mair F, Spiegel W (2014). Communication in cross-cultural consultations in primary care in Europe: the case for improvement. The rationale for the RESTORE FP 7 project. Prim Health Care Res Dev.

[2] Bundesamt für Statistik. Bevölkerungsdaten im Zeitvergleich. In: Bundesamt für Statistik (BFS); 2016. https://www.bfs.admin.ch/bfs/de/home/statistiken/kataloge-datenbanken/tabellen.assetdetail.3442531.html. Accessed 02 July 2018.

[3] Schuster S. Migration und Gesundheit: Diversität und Chancengleichheit am USB. Warum ein solches Projekt am Universitätsspital Basel? Zweizwölf. In: Gazetta des Universitätsspital Basels; 2012. https://www.gazzetta-online.ch/assets/uploads/gazzetta_archiv/2012_2.pdf. Accessed 02 July 2018.

[4] Meissner F, Vertovec S (2015). Comparing super-diversity. Ethnic Racial Stud.

[5] Vertovec S (2007). Super-diversity and its implications. Ethnic Racial Stud..

[6] Bundesamt für Gesundheit (2012). Gesundheit der Migrantinnen und der Migranten in der Schweiz - Wichtigste Ergebnisse des zweiten Gesundheitsmonitorings der Migrationsbevölkerung in der Schweiz, 2010.

[7] Hirsh AT, Hollingshead NA, Ashburn-Nardo L, Kroenke K (2015). The interaction of patient race, provider bias, and clinical ambiguity on pain management decisions. J Pain.

[8] Hurst SA, Forde R, Reiter-Theil S, Slowther AM, Perrier A, Pegoraro R (2007). Physicians' views on resource availability and equity in four European health care systems. BMC Health Serv Res.

[9] Hyatt A, Lipson-Smith R, Schofield P, Gough K, Sze M, Aldridge L (2017). Communication challenges experienced by migrants with cancer: a comparison of migrant and English-speaking Australian-born cancer patients. Health Expect.

[10] Malmusi D, Borrell C, Benach J (2010). Migration-related health inequalities: showing the complex interactions between gender, social class and place of origin. Soc Sci Med.

[11] Marmot M, Allen J, Bell R, Bloomer E, Goldblatt P, CftERoSDoH D (2012). WHO European review of social determinants of health and the health divide. Lancet.

[12] Hudelson P, Vilpert S (2009). Overcoming language barriers with foreign-language speaking patients: a survey to investigate intra-hospital variation in attitudes and practices. BMC Health Serv Res.

[13] Divi C, Koss RG, Schmaltz SP, Loeb JM (2007). Language proficiency and adverse events in US hospitals: a pilot study. Int J Qual Health Care.

[14] Meeuwesen L (2012). Language barriers in migrant health care: a blind spot. Patient Educ Couns.

[15] Carrillo JE, Green AR, Betancourt JR (1999). Cross-cultural primary care: a patient-based approach. Ann Intern Med.

[16] Schouten BC, Meeuwesen L (2006). Cultural differences in medical communication: a review of the literature. Patient Educ Couns.

[17] Gilgen D, Maeusezahl D, Salis Gross C, Battegay E, Flubacher P, Tanner M (2005). Impact of migration on illness experience and help-seeking strategies of patients from Turkey and Bosnia in primary health care in Basel. Health Place.

[18] Napier AD, Ancarno C, Butler B, Calabrese J, Chater A, Chatterjee H (2014). Culture and health. Lancet.

[19] Kleinman A, Benson P (2006). Anthropology in the clinic: the problem of cultural competency and how to fix it. PLoS Med.

[20] CNE, NEK (2017). Migrants allophones et système de soins.

[21] Bundesamt für Gesundheit (2002). Migration und Gesundheit. Strategische Ausrichtung des Bundes 2002–2006.

[22] Bundesamt für Gesundheit (2007). Bundesstrategie "Migration und Gesundheit 2008–2013".

[23] Bundesamt für Gesundheit. Nationales Programm Migration und Gesundheit 2014–2017. Bundesamt für Gesundheit (BAG). 2014. https://www.bag.admin.ch/bag/de/home/themen/strategien-politik/nationale-gesundheitsstrategien/gesundheitliche-chancengleichheit/programm-migration-und-gesundheit-2002-2017.html. Accessed 02 July 2018.

[24] Saba GW, Wong ST, Schillinger D, Fernandez A, Somkin CP, Wilson CC (2006). Shared decision making and the experience of partnership in primary care. Ann Fam Med.

[25] Salmon P, Mendick N, Young B (2010). Integrative qualitative communication analysis of consultation and patient and practitioner perspectives: towards a theory of authentic caring in clinical relationships. Patient Educ Couns.

[26] Bugge C, Entwistle VA, Watt IS (2006). The significance for decision-making of information that is not exchanged by patients and health professionals during consultations. Soc Sci Med.

[27] Reiter-Theil S (2012). What does empirical research contribute to medical ethics? A methodological discussion using exemplary studies. Camb Q Healthc Ethic..

[28] Pope C (2005). Conducting ethnography in medical settings. Med Educ.

[29] Holloway I, Wheeler S (1997). Qualitative Pflegeforschung: Grundlagen qualitativer Ansätze in der Pflege. Reihe Pflegeforschung.

[30] DeWalt KM, DeWalt BR (2002). Participant observation: a guide for fieldworkers.

[31] Altorfer A, Domenig D (2007). Transkulturelle Kompetenz: Lehrbuch für Pflege-, Gesundheits- und Sozialberufe.

[32] Mayring P. Einführung in die qualitative Sozialforschung : eine Anleitung zu qualitativem Denken. 5., überarb. und neu ausgestattete Aufl. ed. Beltz Studium Basel: Beltz; 2002.

[33] Mayring P. Qualitative content analysis: theoretical foundation, basic procedures and software solution. Klagenfurt: 2014. http://nbn-resolving.de/urn:nbn:de:0168-ssoar-395173.

[34] Mayring P (2007). On Generalization in Qualitatively Oriented Research [23 paragraphs]. Forum Qualitative Sozialforschung / Forum: Qualitative Social Research.

[35] Prochaska JO (2008). Decision making in the transtheoretical model of behavior change. Med Decis Mak.

[36] Prochaska JO, DiClemente CC (1982). Transtheoretical therapy: toward a more integrative model of change. Psychotherapy.

[37] Emmerling ME, Whelton WJ (2009). Stages of change and the working alliance in psychotherapy. Psychother Res.

[38] Quenzel G, Schaeffer D, Messer M, Vogt D (2015). Health literacy among less well-educated young people: influencing factors and consequences. Bundesgesundheitsblatt Gesundheitsforschung Gesundheitsschutz..

[39] van Ryn M, Burke J (2000). The effect of patient race and socio-economic status on physicians' perceptions of patients. Soc Sci Med.

[40] Spoont M, Nelson D, van Ryn M, Alegria M (2017). Racial and ethnic variation in perceptions of VA mental health providers are associated with treatment retention among veterans with PTSD. Med Care.

[41] Biyikli Gültekin E. Difficulties in health care for female Turkish immigrants with type 2 diabetes: a qualitative study in Vienna. Wien Klin Wochenschr 2017. doi:10.1007/s00508-017-1190-2. 10.1007/s00508-017-1190-2 28382526

[42] Priebe S, Sandhu S, Dias S, Gaddini A, Greacen T, Ioannidis E (2011). Good practice in health care for migrants: views and experiences of care professionals in 16 European countries. BMC Public Health.

[43] Betancourt JR, Green AR, Carrillo JE, Ananeh-Firempong O (2003). Defining cultural competence: a practical framework for addressing racial/ethnic disparities in health and health care. Public Health Rep.

[44] Ilkilic I (2008). Cultural aspects of ethical decisions at the end of life and cultural competence. Bundesgesundheitsblatt Gesundheitsforschung Gesundheitsschutz.

[45] Brunger F (2016). Guidelines for teaching cross-cultural clinical ethics: critiquing ideology and confronting power in the Service of a Principles-Based Pedagogy. J Bioeth Inq.

[46] Minkoff H (2014). Teaching ethics: when respect for autonomy and cultural sensitivity collide. Am J Obstet Gynecol.

[47] Langewitz W (2007). Beyond content analysis and non-verbal behaviour - what about atmosphere? A phenomenological approach. Patient Educ Couns.

[48] Fonagy P, Gergely G, Jurist E, Target M (2002). Affect regulation, mentalization and the development of the self.

[49] Ahola-Launonen J (2015). The evolving idea of social responsibility in bioethics a welcome trend. Camb Q Healthc Ethic.

[50] Cohen JJ (2006). Professionalism in medical education, an American perspective: from evidence to accountability. Med Educ.

[51] Polit DF, Beck CT (2010). Generalization in quantitative and qualitative research: myths and strategies. Int J Nurs Stud.

